# Genetic Diversity Relationship Between Grain Quality and Appearance in Rice

**DOI:** 10.3389/fpls.2021.708996

**Published:** 2021-08-02

**Authors:** Hua Zhong, Shuai Liu, Gangqing Zhao, Chenhao Zhang, Zhaohua Peng, Zhaohai Wang, Jing Yang, Yangsheng Li

**Affiliations:** ^1^State Key Laboratory of Hybrid Rice, Key Laboratory for Research and Utilization of Heterosis in Indica Rice, Ministry of Agriculture, College of Life Sciences, Wuhan University, Wuhan, China; ^2^Department of Biochemistry, Molecular Biology, Entomology and Plant Pathology, Mississippi State University, Starkville, MS, United States; ^3^Key Laboratory of Crop Physiology, Ecology and Genetic Breeding, Ministry of Education, Jiangxi Agricultural University, Nanchang, China; ^4^College of Life Sciences, Nanchang University, Nanchang, China

**Keywords:** *Oryza sativa* L., gelatinization temperature, amylose content, grain protein content, pericarp color, length-width ratio, grain volume, GWAS

## Abstract

Grain quality is an important breeding objective in rice, and the appearance of the grain also affects its commercial value in the market. The aim of this study was to decode the rice grain qualities and appearances, such as gelatinization temperature (GT), amylose content (AC), grain protein content (GPC), pericarp color (PC), length/width ratio (LWR), and grain volume (GV) at phenotypic and genetic levels, as well as the relationships among them. A genome-wide association study (GWAS) was used to identify the quantitative trait locus (QTLs) associated with the target traits using mixed linear model (MLM) and Bayesian-information and linkage-disequilibrium iteratively nested keyway (BLINK) methods. In general, AC was negatively correlated with GPC and GV, while it was positively correlated with LWR and PC. GPC was positively correlated with LWR. Using the rice diversity panel 1 (RDP1) population, we identified 11, 6, 2, 7, 11, and 6 QTLs associated with GT, AC, GPC, PC, LWR, and GV, respectively. Five germplasm lines, superior in grain qualities and appearances for basic breeding materials or improvement, were identified. Notably, an F-box gene *OsFbox394* was located in the linkage disequilibrium (LD) block of *qLWR7-2*, which specifically expresses in endosperm and seed tissues, suggesting that this gene may regulate the seed development in rice grain. Besides, different haplotypes of *OsHyPRP45* showed significant differences in AC, indicating that this gene may be related to AC in rice grain.

## Introduction

Rice (*Oryza sativa* L.) is one of the most widely cultivated cereal crops all over the world and provides the staple food for over half of the world population (Mbanjo et al., 2020). With modern technology advancement and improvement of the quality of life, people are seeking food with high nutritional and appearance qualities. Eating and cooking qualities (ECQs) and grain protein content (GPC) are the main factors that determine rice grain quality. ECQ could be further dissected into amylose content (AC), gel consistency (GC), and gelatinization temperature (GT). The AC could be divided into five groups, namely, waxy (0–2%), very low (3–9%), low (10–19%), intermediate (20–25%), and high (>25%). Rice grains with an AC of 16–20% are the most popular type in markets and meet the demand of ECQ from customers (Song et al., 2019). GT is usually measured by alkali spreading value (ASV), which is evaluated by the extent of dispersal of whole milled rice grains in a dilute alkali solution (Pang et al., 2016). GPC is the most vital nutritional compound in rice grain around 8%, which is lower than other cereal grains (Chen et al., 2018). Glutelin, albumin, globulin, and prolamin are the four components of rice seed storage protein. Rice grain has a higher percentage of glutelin, which is more easily digested by humans, making it a superior resource of high-quality protein. Pericarp color (PC), length/width ratio (LWR), and grain volume (GV) are important features that influence the appearance quality of rice. The red and purple pigments in rice grains are mainly proanthocyanidin and anthocyanidin, respectively. Both of the compounds have antioxidant, antidiabetic, antihyperlipidemic, and anticancer activities, which benefit human health (Mbanjo et al., 2020). The volume and shape of grain are key factors that determine the rice yield and market value. In general, the slender shape of rice is usually preferred by customers in the United States, Southern China, and South and Southeast Asian countries, while the short and round rice grains cater to the consumers in Northern China, Korea, and Japan (Huang et al., 2013).

Many genes have been identified controlling the rice grain qualities. *OsGBSSI* (Wang et al., 1995) and *OsSSIIa* (Gao et al., 2011) were major genes regulating AC and GT in rice grain, respectively. *OsAAP6* (Peng et al., 2014) and *OsGluA2* (Yang et al., 2019) were two cloned genes that determine the GPC in rice. *GS3* (Fan et al., 2006), *qGL3* (Zhang et al., 2012), *GL7* (Wang et al., 2015), *GW2* (Song et al., 2007), *GW5* (Weng et al., 2008), *GS5* (Xu et al., 2015), *qTGW3* (Hu et al., 2018), and *GW8* (Wang et al., 2012) were key regulators of the shape and size of the rice grain, controlling the LWR, GV, and grain weight. *Rc, Rd* (Furukawa et al., 2007), and *OsF3H2* (Wang et al., 2016) were genes reported related to PC in rice grain.

In a previous study, 44k single nucleotide polymorphism (SNP) variants were used to identify 34 QTLs in rice diversity panel 1 (RDP1) accessions (Zhao et al., 2011). The release of the High-Density Rice Array (HDRA), containing 700k SNP (McCouch et al., 2016), allows us to map more precisely for certain traits in the RDP1 population. In this study, the distribution and relationships among AC, GT, GPC, PC, LWR, and GV were analyzed, and two genome-wide association study (GWAS) methods (i.e., MLM and BLINK) were used to identify the QTLs associated with the target traits. Superior lines for high GPC and satisfactory ECQ were selected for breeding improvement. In addition, candidate genes controlling AC and LWR in rice were detected, and further experiments were required for validating the function of candidate genes.

## Materials and Methods

### Plant Materials

The RDP1 consists of 421 purified homozygous varieties (Eizenga et al., 2014) such as indica (IND), aus (AUS), tropical japonica (TRJ), temperate japonica (TEJ), and aromatic subgroups (ARO). Among them, 406 have been genotyped by the HDRA, which is used in this study. The detailed information of the varieties is listed in Supplementary Table 1.

### Determination of the Grain Quality and Appearance Quality

Six traits that were widely used to characterize rice grain quality and apparent traits were studied in this study, such as grain amylose content (AC), alkali spreading value (ASV), grain protein content (GPC), grain length/width ratio (LWR), grain volume (GV), and pericarp color (PC). All the traits were obtained from the USDA website (https://www.ars.usda.gov/southeast-area/stuttgart-ar/dale-bumpers-national-rice-research-center/docs/rice-diversity-panel-1-rdp1/).

### SNP Data Set and Population Structure

The HDRA (700k SNPs) file was downloaded from the Rice Diversity website (http://www.ricediversity.org/data/). The detailed information of samples used in this study was listed in Supplementary Table 1. The same procedure was used to preprocess the genotype data set with a previous study (Zhong et al., 2021).

### Genome-Wide Association Study

The GWAS was performed among 406 rice varieties derived from RDP1 with 411,066 high-quality SNPs. Two GWAS methods (i.e., MLM and BLINK) were employed to evaluate the trait–SNP associations for grain quality and appearance traits using the Genomic Association and Prediction Integrated Tool (GAPIT) (Lipka et al., 2012). The first four principal components (PCs) were used as covariates to correct population structure due to population stratification in RDP1. To control the Type I error (i.e., false-positive), we set *p*-value = 2.20E-07 as a threshold, which was determined by 0.05/*n*, where *n* is the effective number of independent markers. The effective number of independent markers (*n* = 227,753) was calculated using GEC software version 0.2 (Li et al., 2012). The Manhattan and Q–Q plots for GWAS were visualized using the R package *qqman* (D. Turner, 2018).

### Mining Candidate Genes and Annotation of SNPs

The QTLs identified that the MLM and BLINK models provide important information for understanding the genetic architecture of grain quality and appearance in rice. To explore candidate genes responsible for each QTL, we defined local LD with the CI method (Gabriel et al., 2002), and all genes in the blocks were extracted for further analysis. For the QTLs that failed to define LD blocks, we extracted genes in the 100 kb upstream and downstream of leading SNPs. The gene annotation file was downloaded from The Rice Annotation Project Database website (https://rapdb.dna.affrc.go.jp/index.html). Then, the SnpEff software (Cingolani et al., 2012) was used to annotate the effect of the variant for candidate genes.

### Gene Expression Analysis

The gene expression profile of candidate gene *OsFbox394* in 9 tissues, seed, endosperm, embryo, anther, pistil, pre-inflorescence, pre-inflorescence, shoot, and leaves, was downloaded from Rice Genome Annotation Project (http://rice.plantbiology.msu.edu/expression.shtml). The expression data of *OsHyPRP45* in 36 tissues in three cultivars (i.e., Minghui 63, Shanyou 63, and Zhenshan 97) had been harvested from the CREP database (http://crep.ncpgr.cn/crep-cgi/). Fragments Per Kilobase Million (FPKM) was used to represent the gene expression levels.

## Results

### Grain Quality and Apparent Variant Among the Rice Subpopulations

**Figure 1:**
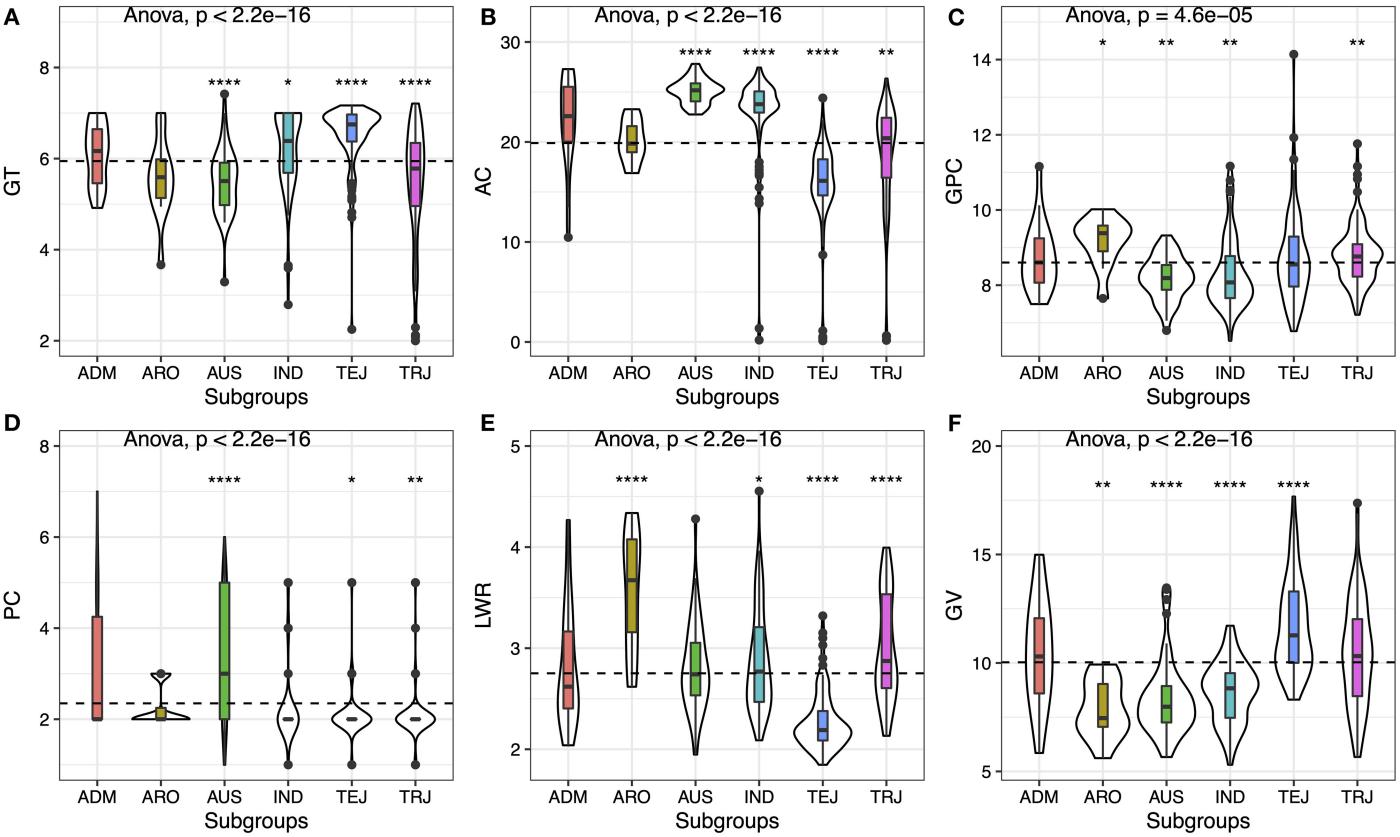
Phenotypic variation of six traits among the rice subgroups. **(A)** Gelatinization temperature (GT), **(B)** amylose content (AC), **(C)** grain protein content (GPC), **(D)** pericarp color (PC), **(E)** length/width ratio (LWR), and **(F)** grain volume (GV). Violin plots show the dispersion and the distribution of the traits in subgroups. Whiskers represent 1.5 times the interquartile of the data. The upper and lower edge of the box presents the interquartile range of the data. The thick black bar in the center represents the median of the data. The one-way ANOVA test is used to determine whether there are any statistically significant differences between the subgroups, and the *p*-value is shown on the left corner of each figure. The total population acts as a reference group, and each subgroup is compared with the reference group. Different symbols above each subgroup indicate significant differences: ^*^p < 0.05, ^**^p < 0.01, ^***^p < 0.001, and ^****^p < 0.0001. The dashed black line represents the mean value of the whole population.

Based on the principal component analysis (PCA), we divided the varieties into six subgroups [i.e., IND, AUS, TEJ, TRJ, ARO, and ADM (Admixture)]. We analyzed the distribution of the six traits in subgroups, and the results showed that all of the traits have significant differences (*p* < 2.2E-16) among the subgroups except protein content, indicating that this trait was less variable compared with other traits (Figure 1). TEJ and IND had higher GT, while TRJ and AUS subgroups exhibited lower GT (Figure 1A). AUS and IND showed higher AC, and especially AUS displayed the highest AC. The AC was different in the *japonica* subspecies (i.e., TRJ and TEJ), and TRJ had relatively higher AC, while TEJ contained the lowest AC (Figure 1B). GPC was one of the most valuable nutrition in rice grain; the ARO subgroups showed the highest mean value of GPC, while AUS and IND subgroups showed relatively lower ingredients (Figure 1C). PC was also another important character in rice. *Japonica* species (i.e., TEJ and TRJ) had a bigger chance to produce white or light brown color rice, while the AUS subgroup usually exhibited darker color (i.e., red or brown) (Figure 1D). By analyzing the seed volume and seed LWR, we found that the ARO subgroup showed a relatively smaller and slender grain, while the TEJ subgroup displayed a bigger and round appearance (Figures 1E,F).

From the abovementioned details, the ARO subgroup contained the highest grain GPC with darker color and slender shape, which could provide both protein and antioxidant compounds. But the seed size was the lowest, and the AC was relatively higher, which was needed to be improved. GV in TEJ was significantly bigger than other subgroups, suggesting some genes may play a key role in the seed development in TEJ. Thus, by mapping the gene associated with AC and GV, we could decrease the AC and increase the GV for the targeting elite varieties from ARO.

### Grain Quality and Apparent Variant Among the Rice Groups From Different Geographic Regions

**Figure 2:**
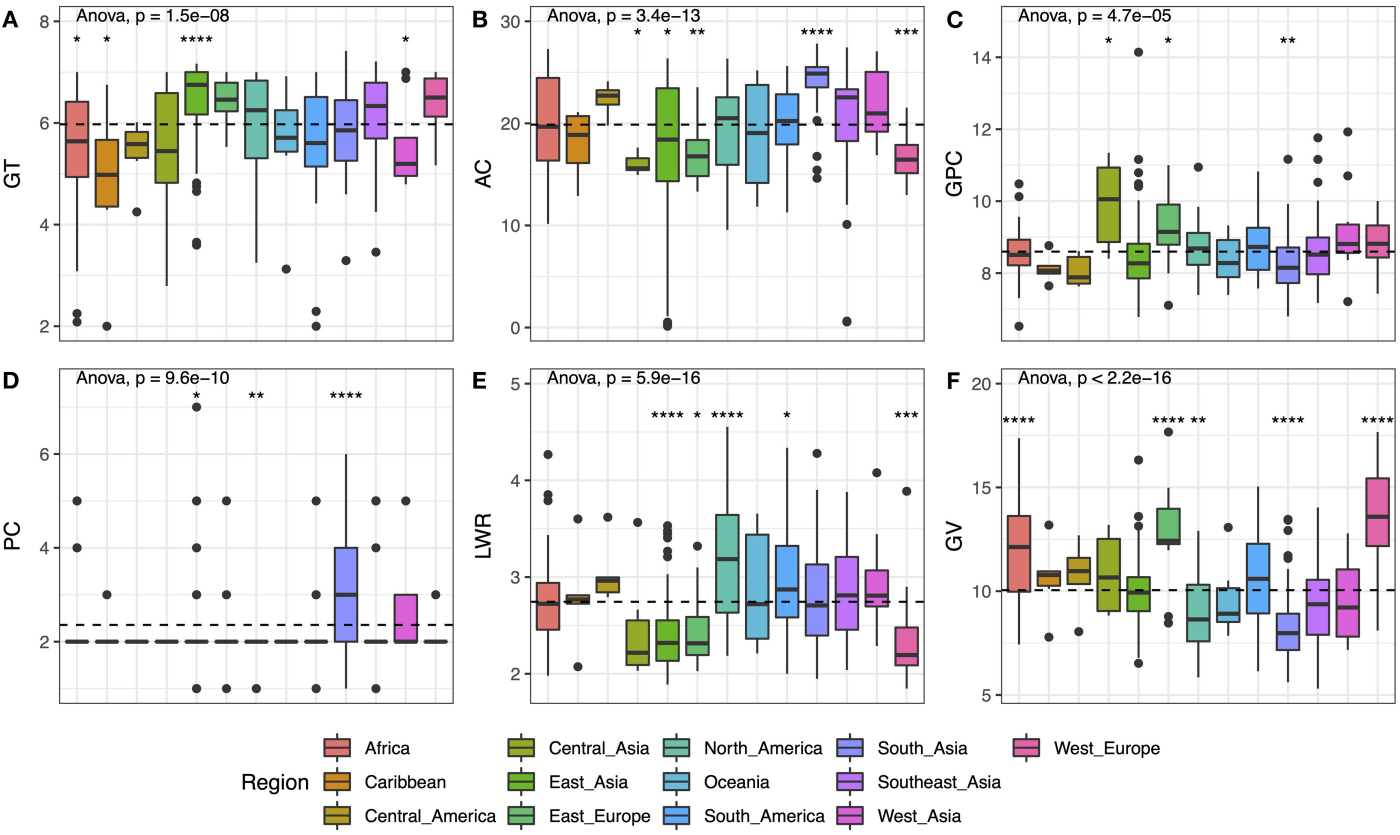
Phenotypic variation of six traits based on different geographical regions. **(A)** GT, **(B)** AC, **(C)** GPC, **(D)** PC, **(E)** LWR, and **(F)** GV. Box plots show the distribution of the traits in subgroups. The numbers of accessions originated from Africa, Caribbean, Central America, Central Asia, East Asia, East Europe, North America, Oceania, South America, South Asia, South Asia, Southeast Asia, West Asia, and West Europe are 40, 7, 7, 7, 85, 16, 46, 7, 32, 68, 51, 12, and 22, respectively. Whiskers represent 1.5 times the interquartile of the data. The upper and lower edge of the box presents the interquartile range of the data. The thick black bar in the center represents the median of the data. The one-way ANOVA test is used to determine whether there are any statistically significant differences between the subgroups, and the *p*-value is shown on the left corner of each figure. The total population acts as a reference group, and each subgroup is compared with the reference group. Different symbols above each subgroup indicate significant differences: ^*^p < 0.05, ^**^p < 0.01, ^***^p < 0.001, and ^****^p < 0.0001. The dashed black line represents the mean value of the whole population.

The 406 varieties studied in this study are from 13 regions all over the world. The detailed information was listed in Supplementary Table 1. By analyzing the distribution of the six traits in subgroups, the results showed significant differences in all populations from *p*-value = 4.7E-05 (GPC) to *p*-value <2.2E-16 (GV) (Figure 2). The materials from East Asia, East Europe, and West Europe showed higher GT with lower AC, and most of them were round in shape. While the GV among the three areas was significantly different, the seed size from Europe (i.e., East Europe and West Europe) exhibits an obvious bigger compared with that from East Asia. Besides, the seeds from Central Asia present the highest mean value of GPC with proper AC (with the median at 15.60%), but the grain size of Central Asia was not the largest, which was need to be improved for increasing yield. The varieties from South Asia contained lower GPC, smallest GV, and highest AC, which seemed to be not the ideal basic materials for breeding. But the color of the materials from South Asia was relatively darker compared with other regions, meaning more pigment accumulation in these seeds. Thus, these seeds were good materials for us to study the genes regulating grain color and unitized to enhance the antioxidant activities in elite cultivars.

### Variation of Grain Quality in Relationship With Apparent Traits

**Figure 3:**
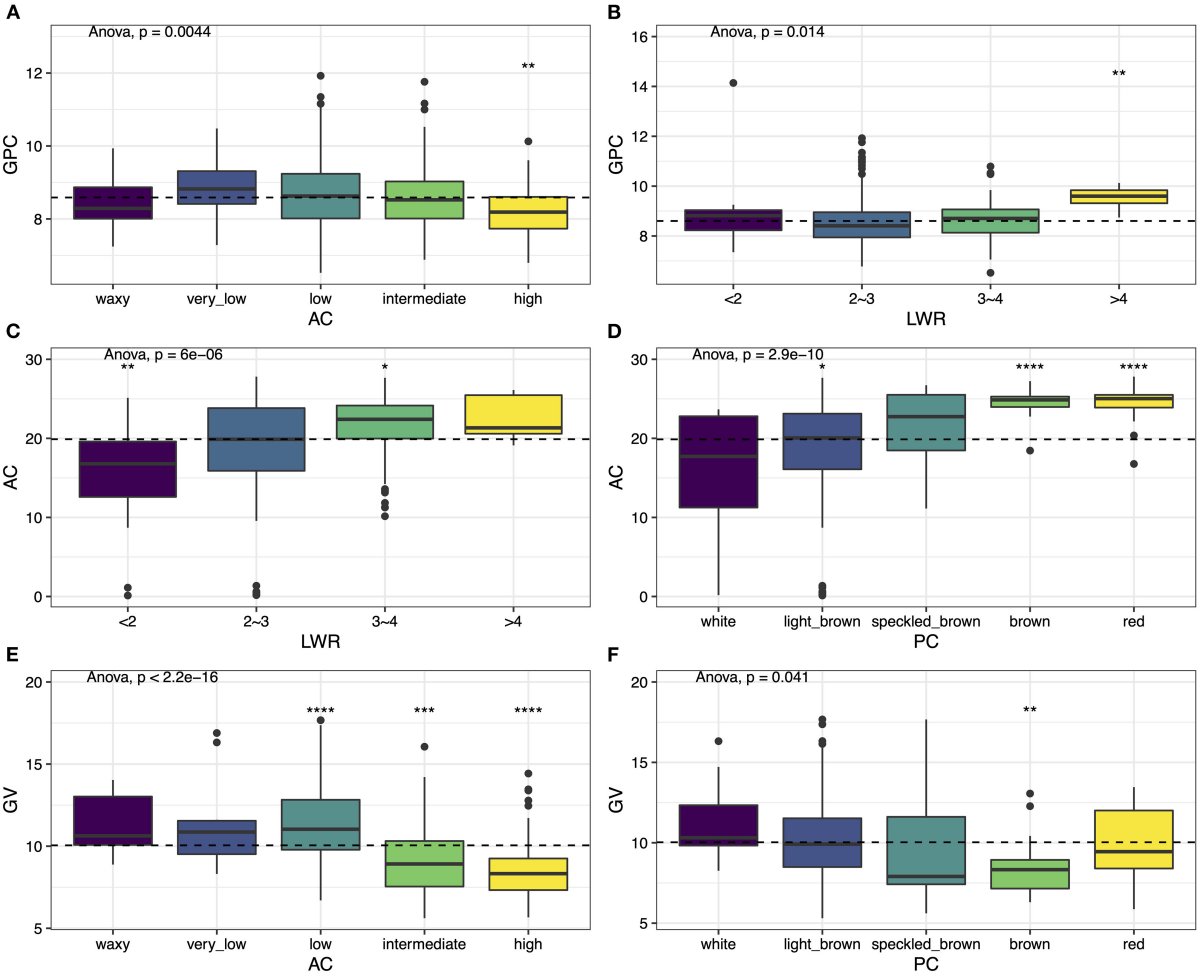
Phenotypic variation among six traits. **(A)** GPC vs. AC, **(B)** GPC vs. LWR, **(C)** AC vs. LWR, **(D)** AC vs. PC, **(E)** GV vs. AC, and **(F)** GV vs. PC. Box plots show the distribution of the traits in subgroups. Whiskers represent 1.5 times the interquartile of the data. The upper and lower edge of the box presents the interquartile range of the data. The thick black bar in the center represents the median of the data. The one-way ANOVA test is used to determine whether there are any statistically significant differences between the subgroups, and the *p*-value is shown on the left corner of each figure. The total population acts as a reference group, and each subgroup is compared with the reference group. Different symbols above each subgroup indicate significant differences: ^*^p < 0.05, ^**^p < 0.01, ^***^p < 0.001, and ^****^p < 0.0001. The dashed black line represents the mean value of the whole population.

We compared the GPC among the differences of AC and LWR. In general, the GPC varies along with the AC and the LWR. The seed that contained the highest GPC usually exhibited very low AC (Figure 3A) and slender seed shape (Figure 3B). The grain with higher AC usually had the potential for the storage of lower GPC. The intermediate type of seed shape (i.e., LWR with 2–3) contained the lowest GPC. The relationship of AC with LWR and PC was also performed. The results showed that the seeds with a slender shape (Figure 3C) and a darker color (Figure 3D) usually present higher AC. The darker the PC, the higher the AC in rice grain. The round-shaped and white color rice had the proper AC, which was the most favorite type in customers. Finally, we also compared the GV differences between AC and PC. The seeds with low AC had the performance of the largest size of rice grain (Figure 3E), and the smaller size of rice grain usually exhibited higher AC (Figure 3E). PC also represents differences with seed volume on the overall population level (Figure 3F). The white materials exhibited a bigger size compared with colored rice, and the brown color rice was the smallest in the current population. The Spearman's correlation analysis was also performed to study the relationship among the traits, and similar results were obtained (Supplementary Table 2).

### Genome-Wide Association Study of Grain Quality and Apparent Traits

**Figure 4:**
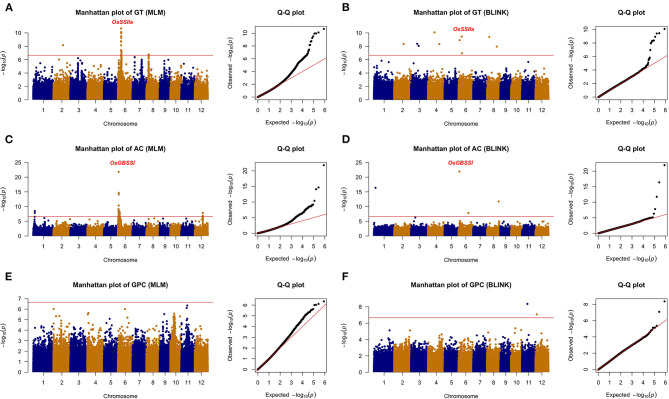
The genome-wide association analysis of grain quality with MLM and BLINK methods. **(A)** GT with MLM, **(B)** GT with BLINK, **(C)** AC with MLM, **(D)** AC with BLINK, **(E)** GPC with MLM, and **(F)** GPC with BLINK. The horizontal red line indicated the significance thresholds at –log10(*p*) = 6.66.

**Figure 5:**
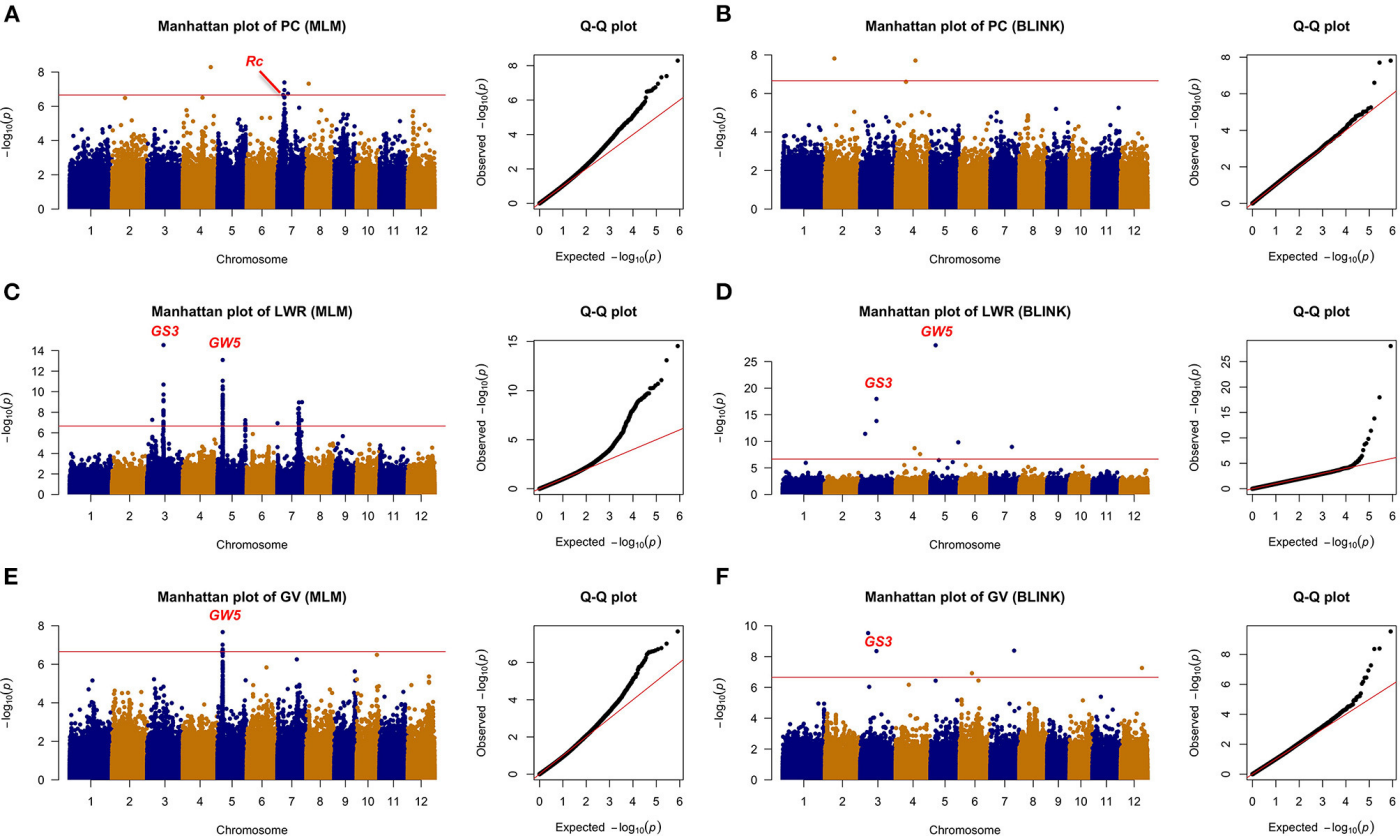
The genome-wide association analysis of appearance with MLM and BLINK methods. **(A)** PC with MLM, **(B)** PC with BLINK, **(C)** LWR with MLM, **(D)** LWR with BLINK, **(E)** GV with MLM, and **(F)** GV with BLINK. The horizontal red line indicated the significance thresholds at –log10(*p*) = 6.66.

All the six traits were analyzed using the two GWAS models (i.e., MLM and BLINK) to identify QTLs. Both the PCA and relatedness matrixes were incorporated in the MLM model to reduce the false-positive rate. BLINK model approximates the maximum likelihood using Bayesian Information Criterion in a fixed-effect model to reduce the amount of calculation. Specifically, 10, 6, 2, 7, 11, and 6 loci were detected associated with GT, AC, GPC, PC, LWR, and GV, respectively (Table 1; Figures 4, 5).

**Table 1:** Significant SNP associated for six traits detected by genome-wide association study (GWAS) with MLM and BLINK models.

**Trait**	**QTL**	**Chromosome**	**Position**	***p*-Value from MLM**	***p*-Value from BLINK**	**Reported genes**
GT	*qGT2-1*	2	20,220,266	6.90E-09	4.69E-09	
	*qGT3-1*	3	13,235,693		4.22E-09	
	*qGT3-3*	3	16,312,643		8.62E-09	
	*qGT4-1*	4	13,968,254		8.32E-11	
	*qGT4-2*	4	24,196,718		4.81E-09	
	*qGT6-1*	6	2,195,964		1.21E-09	
	*qGT6-2*	6	6,687,883	2.15E-11	3.52E-10	*OsSSIIa* Gao et al. (2011)
	*qGT6-3*	6	6,830,286	7.20E-09		
	*qGT6-4*	6	7,024,152		1.11E-07	
	*qGT8-1*	8	4,750,713		4.01E-10	
	*qGT8-2*	8	20,763,355		1.11E-08	
AC	*qAC1-1*	1	3,184,457	1.48E-08		
	*qAC1-2*	1	3,329,456	3.18E-09	4.08E-17	
	*qAC6-1*	6	1,765,761	1.57E-22	1.19E-22	*OsGBSSI* Wang et al. (1995)
	*qAC6-2*	6	20,854,368		1.65E-08	
	*qAC8-1*	8	24,785,382		1.82E-12	
	*qAC12-1*	12	17,068,981	1.42E-08		
GPC	*qGPC11-1*	11	11,666,233		4.38E-09	
	*qGPC12-1*	12	2,673,221		8.35E-08	
PC	*qPC2-1*	2	9,655,892		1.53E-08	
	*qPC4-1*	4	20,157,178		1.96E-08	
	*qPC4-2*	4	28,610,659	5.14E-09		
	*qPC7-1*	7	6,068,017	2.17E-09		*Rc* Furukawa et al. (2007)
	*qPC7-2*	7	7,239,363	4.06E-08		
	*qPC7-3*	7	10,922,526	1.82E-07		
	*qPC8-1*	8	2,373,426	4.78E-08		
LWR	*qLWR3-1*	3	5,210,399	5.44E-08	3.92E-12	
	*qLWR3-2*	3	16,733,441	2.89E-15	1.05E-18	*GS3* Mao et al. (2010)
	*qLWR3-3*	3	16,749,510		1.50E-14	
	*qLWR4-1*	4	19,267,463		1.95E-09	
	*qLWR4-2*	4	24,917,693		2.56E-08	
	*qLWR5-1*	5	5,371,772	8.18E-14	8.50E-29	*GW5* Weng et al. (2008)
	*qLWR5-2*	5	28,480,050	5.96E-08	1.53E-10	
	*qLWR5-3*	5	28,487,739	1.21E-07		*OsRPH1* Ma et al. (2020)
	*qLWR7-1*	7	217,125	1.17E-07		
	*qLWR7-2*	7	22,113,010	1.10E-09	1.11E-09	
	*qLWR7-3*	7	25,214,651	1.03E-09		
GV	*qGV3-1*	3	8,210,958		2.98E-10	
	*qGV3-2*	3	16,733,441		4.41E-09	*GS3* Mao et al. (2010)
	*qGV5-1*	5	5,355,339	2.13E-08		*GW5* Weng et al. (2008)
	*qGV6-1*	6	12,504,994		1.20E-07	
	*qGV7-1*	7	24,413,754		4.12E-09	
	*qGV12-1*	12	21,773,741		5.48E-08	

*GT, gelatinization temperature; AC, amylose content; GPC, grain protein content; PC, pericarp color; LWR, length/width ratio; and GV, grain volume*.

Among the 10 loci associated with GT, *qGT6-2* located 60 kb away from the reported gene *OsSSIIa*, which was detected by both methods with a significant *p*-value (i.e., 2.15E-11 from MLM and 3.52E-10 from BLINK). The *qGT2-1* was another significant QTL identified with the BLINK and MLM models in Chromosome 2. Eight more loci were detected only by BLINK. For AC, two QTLs were detected by both models. The *qAC6-1* was identified with an extremely high significant *p*-value (i.e., 1.57E-22 from MLM and 1.19E-22 from BLINK), which was nearby a reported gene *OsGBSSI* (Wang et al., 1995). Besides, another novel QTL, i.e., *qAC1-2*, was also detected by both methods. GPC was another important rice grain quality trait, which determined the nutritional value. Only two QTLs (i.e., *qGPC11-1* and *qGPC12-1*) were identified by the BLINK model, while none of them were detected by the MLM model.

A total of 24 loci were detected associated with rice grain appearance traits, with 7, 11, and 6 for PC, LWR, and GV, respectively. Colorless rice is the most consumption type all over the world, while colored rice has been studied and showed its unique activity in reducing the risk of developing chronic diseases, such as cardiovascular disease and Type 2 diabetes (Tantipaiboonwong et al., 2017). Five QTLs (i.e., *qPC4-1, qPC7-1, qPC7-2, qPC7-3*, and *qPC8-1*) and two QTLs (i.e., *qPC2-1* and *qPC4-1*) were detected to be associated with the color of the rice seed by the MLM and BLINK models, respectively, while there was no overlapped QTL between the two models. Notably, the *qPC7-1* was overlapped with the gene *Rc*, which was reported regulating the synthesis of proanthocyanidin pigmentation (Furukawa et al., 2007). Different shapes of rice cater to various consumers all over the world. We detected 11 loci tightly associated with rice shape in this study. Five of them (i.e., *qLWR3-1, qLWR3-2, qLWR5-1, qLWR5-2*, and *qLWR7-2*) were detected by both models simultaneously. Seed size (GV) was an important factor related to the yield. The bigger size of rice grain was one of the breeding strategies to improve the rice yield. Six QTLs were detected in this study with two methods. The *qGV3-2* was identified by the BLINK method with a significant *p*-value of 4.41E-09, while *qGV5-1* was detected by the MLM model. Four more significant loci were identified on Chromosome 3, 6, 7, 12, respectively.

**Figure 6:**
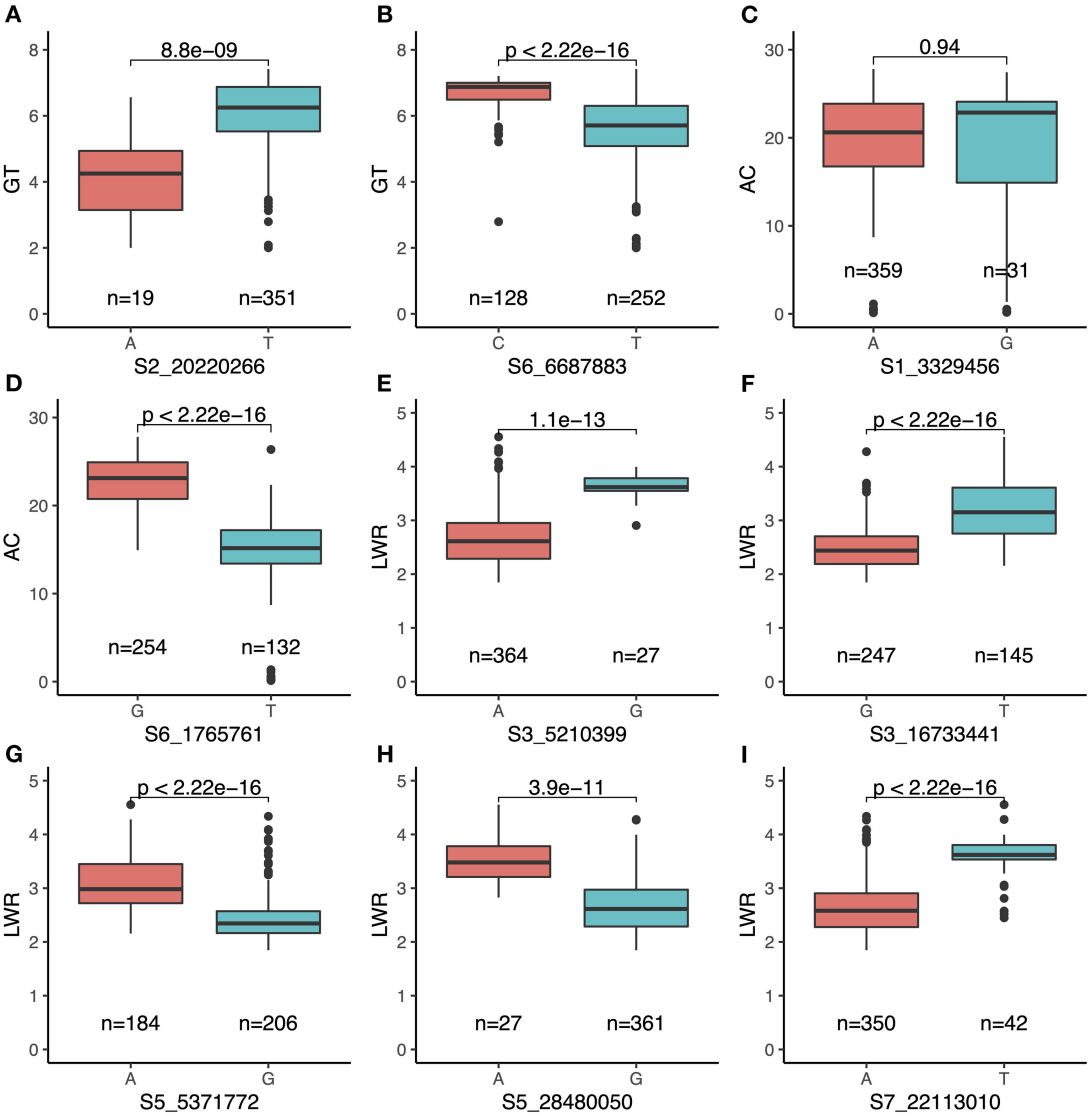
Comparison of nine QTLs simultaneously identified by both MLM and BLINK carrying major and minor alleles of each SNP associated with the traits. **(A)** *qGT2-1* and **(B)** *qGT6-2* for GT; **(C)** *qAC1-2* and **(D)** *qAC6-1* for AC; **(E)** *qLWR3-1*, **(F)** *qLWR3-2*, **(G)** *qLWR5-1*, **(H)** *qLWR5-2*, and **(I)** *qLWR7-2* for LWR.

Above all, a total of nine QTLs (*qGT2-1, qGT6-2, qAC1-2, qAC6-1, qLWR3-1, qLWR3-2, qLWR5-1, qLWR5-2*, and *qLWR7-2*) were simultaneously identified by the MLM and BLINK methods associated with GT, AC, and LWR. To further study the favorable SNPs in each QTL, we compared the distribution of leading SNPs. All the results showed significance except *qAC1-2* (Figure 6). The *qGT2-1* showed a significant association with GT. The minor allele (i.e., the second most common allele, A) of SNP S2_20220266 showed significantly lower GT compared with the major allele (T) in the whole population (Figure 6A). Besides, the minor allele of S2_20220266 was only found in TEJ and TRJ. The individuals with A had significantly lower GT compared with T from TRJ (Supplementary Figure 2A). The *qAC1-2* was identified in association with AC using both the MLM and BLINK methods. The minor allele (G) of SNP S1_3329456 did not show significant differences of GT compared with major allele (A) in the whole population (Figure 6C). Then, we compared the two alleles in the subpopulations, and the samples with minor allele (G) exhibited significantly lower AC compared with major allele (A) in the IND subpopulation (Supplementary Figure 2B). A total of three novel QTLs (i.e., *qLWR3-1, qLWR5-2*, and *qLWR7-2*) were simultaneously associated with LWR in this study. All the leading SNPs of these QTLs displayed significant differences in the whole population (Figures 6E,H,I). Of the 27 accessions carrying minor alleles (G) of SNP S3_5210399, i.e., for SNP S3_5210399, 25 were from TRJ, which was supposed to contribute to slender seed shape (Supplementary Figure 2C). Similarly, minor alleles (A) of SNP S5_28480050 contributed to a slimmer shape of rice in ARO and IND subgroups (Supplementary Figure 2D). Besides, a total of 42 minor alleles (T) were identified in S7_22113010 contributing to LWR, and the minor allele existed in AUS, IND, and TRJ subgroups (Supplementary Figure 2E).

### Mining Candidate Genes for Each QTL

All the local LD blocks were defined for each QTL (Supplementary Table 3). Then, the genes in the LD blocks were extracted for further study (Supplementary Table 4). In the local LD blocks, several genes were remapped for target traits, such as *OsGBSSI* (*Os06g0133000*) for AC, *Rc* (*Os07g0211500*) for PC, *GS3* (*Os03g0407400*) for LWR and GV, and *GW5* (*Os05g0187500*) for LWR and GV. Besides, we also identified missense SNPs in these genes, which corresponded to previous studies (Supplementary Table 5). All the detailed information was listed in Supplementary Table 5.

**Figure 7:**
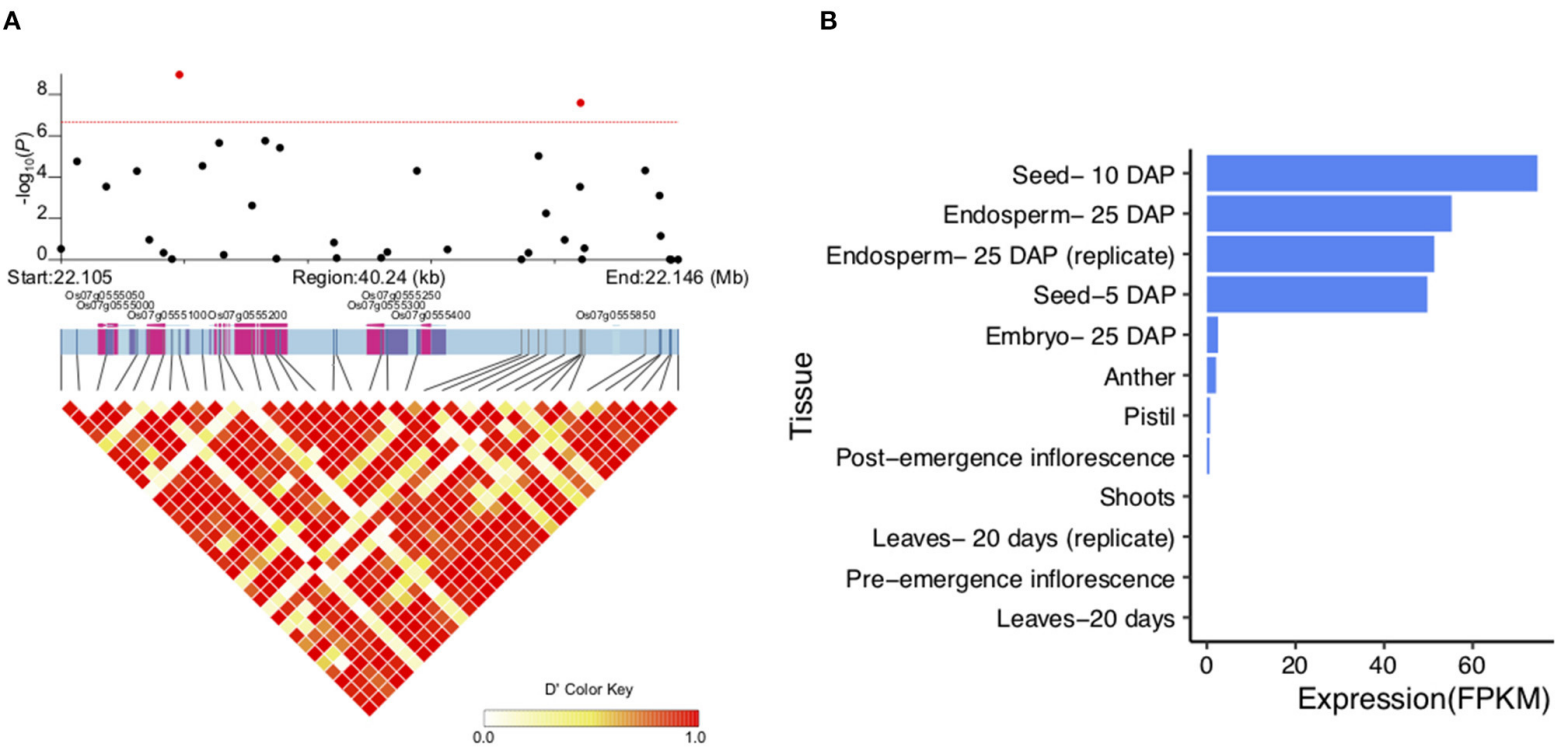
Candidate genes in *qLWR7-2*. **(A)** Local LD block of *qLWR7-2*. Pink color represents the exons, and purple color represents untranslated region (UTR). **(B)** Expression levels of *Os07g0555000* in 9 rice tissues.

**Figure 8:**
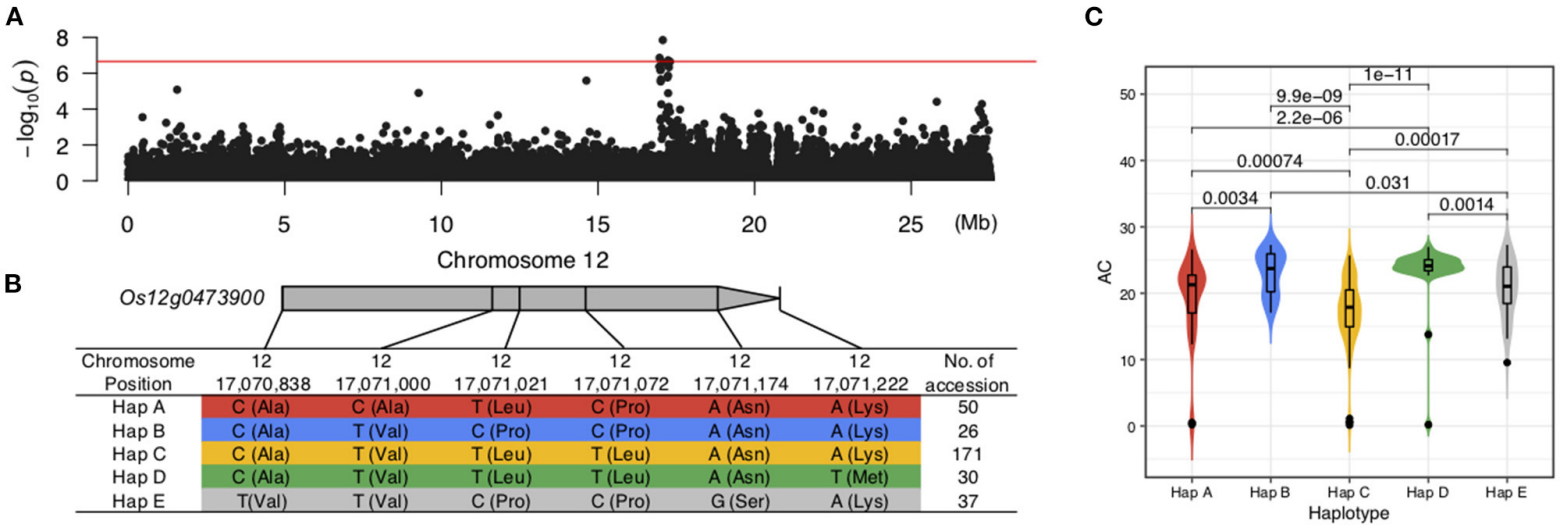
Candidate genes in *qAC12-1*. **(A)** Manhattan plot of AC on Chromosome 12. **(B)** Five haplotypes were defined by six missense SNPs in *Os12g04739900*. **(C)** Significant differences were identified among the five haplotypes from *Os12g04739900*.

Among the QTLs associated with LWR, the *qLWR7-2* was identified simultaneously by both the MLM and BLINK methods with a significant *p*-value of 1.10E-09 and 1.11E-09, respectively. Then, a 40.24-kb block was defined (Figure 7A; Supplementary Table 3), and only eight genes (Table 2) were located in this region. Six of them were unknown genes, and *Os07g0555200* was a translation initiation factor 4G, which resisted to rice tungro spherical virus. Notably, the *OsFbox394* (*Os07g0555000*) was an F-box domain protein. F-box was a big gene family in rice containing 687 members (Jain et al., 2007), which played a crucial role in several biological processes, such as flower development (Duan et al., 2012), leaf senescence (Chen et al., 2013), grain size (Chen et al., 2013), and the development of inflorescence branches and spikelets (Ikeda et al., 2007). In this study, no missense SNP was found in the CDS region of this gene. Then, we investigated the gene expression levels in 9 tissues, and the results showed *OsFbox394*, which is specifically expressed in rice seed and endosperm tissues (Figure 7B), suggesting this gene might be a regulator in seed development by manipulating the expressing level. The *qAC12-1* was a significant (*p*-value = 1.42E-08) QTL associated with AC identified by the MLM method (Figure 8A). Only four genes were located in the local LD block of *qAC12-1*, namely, *Os12g0472500, Os12g0472800, Os12g0472900*, and *Os12g0473900*. Among them, *OsHyPRP45* (*Os12g0473900*) was annotated as a protease inhibitor/seed storage/lipid transfer proteins family (Supplementary Table 4). The tissue-specific expression analysis revealed that the expression levels of *OsHyPRP45* were enhanced in rice seeding (i.e., three days after sowing) and plumule (i.e., 48 h after emergence) (http://ricevarmap.ncpgr.cn/vars_in_gene/). Five haplotypes were defined based on the six missense SNPs in this gene (Figure 8B), and Hap C showed significantly lower AC compared with Hap B (*p*-value = 9.9E-09), Hap D (*p*-value = 1E-11), etc., (Figure 8C). Above all, we suggested *OsHyPRP45* was a candidate gene regulating AC in rice grain.

**Table 2:** Candidate genes located in the local LD block of qLWR7-2.

**Gene name**	**Chromosome**	**Start**	**End**	**Direction**	**Annotation**
*Os07g0555000*	7	22,107,689	22,110,130	+	F-box domain, Skp2-like domain-containing protein.
*Os07g0555050*	7	22,107,715	22,108,709	–	Hypothetical protein.
*Os07g0555100*	7	22,110,826	22,113,674	+	Conserved hypothetical protein.
*Os07g0555200*	7	22,114,961	22,123,129	+	Translation initiation factor 4G, resistance to Rice tungro spherical virus.
*Os07g0555250*	7	22,125,123	22,129,938	–	Non-protein coding transcript.
*Os07g0555300*	7	22,125,218	22,127,923	+	Conserved hypothetical protein.
*Os07g0555400*	7	22,128,502	22,130,398	+	Conserved hypothetical protein.
*Os07g0555850*	7	22,141,246	22,141,737	+	Non-protein coding transcript.

## Discussion

A total of 406 accessions were analyzed in this study, which exhibited a diversity of grain quality and appearance. The GT of IND, TEJ, and TRJ was relatively high, which needs to be reduced for catering customers. Only the accessions from TEJ had the preferred AC, while most of the accessions from other subgroups need to be decreased. The GPC was an important nutritional content in rice grain. In this study, the accessions from ARO or TRJ had higher GPC than others, suggesting we might discover key genes regulating GPC from ARO and TRJ. Purple and red rice grains contain anthocyanins and proanthocyanidins, respectively (Furukawa et al., 2007), which could act as antioxidants (Kong et al., 2003; Rauf et al., 2019) and could benefit human health. The accessions from AUS usually displayed red or purple rice grain, while the majority of individuals from other subgroups showed white or light brown color. The grain size and shape of rice influenced each other, and the accessions with slender shapes frequently had a smaller size, such as those from ARO. In contrast, the round-shaped rice generally exhibited a bigger size, including the accessions from TEJ. These wide variations provide potential in improving both grain quality and appearance simultaneously (Table 3). For example, Ta Mao Tsao (NSFTV155) and Ligerito (NSFTV350) were two accessions with good performances of high GPC, big grain size, desirable GT, and AC, which had the potential of being basic materials for breeding. The Karabaschak (NSFTV224) was a unique material with a large GV, red color, high GPC, and suitable AC with a higher GT, which could be improved in GT by manipulating the genes controlling it. Another accession, WIR 3764 (NSFTV306), with similar characteristics of Karabaschak along with light brown color, was also needed to decrease GT for better ECQs. A possible approach to decrease the GT of NSFTV224 and NSFTV306 is to replace the allele C at the S6_6687883 with the allele G of the *OsSSIIa* gene (Figure 6). A TEJ line, R 101 (NSTFV310), with a high GPC, medium size, suitable GT, and low AC, which could be improved in grain size and AC by the key genes *GS3*, changes allele T at S3_16733441 to allele G (Fan et al., 2006) and *OsGBSSI* (changes allele T at S6_1765761 to allele G) (Wang et al., 1995).

**Table 3:** Germplasm lines selected from the rice diversity panel 1 (RDP1) population in this study.

**NSFTV ID**			**NSFTV155**	**NSFTV224**	**NSFTV306**	**NSFTV310**	**NSFTV350**
GSOR ID			301146	301215	301296	301300	301340
Name			Ta Mao Tsao	Karabaschak	WIR 3764	R 101	Ligerito
Region			East Asia	East Europe	Central Asia	Africa	South America
AC (%)			18.80	16.76	16.87	10.15	16.95
GT			4.82	6.38	6.88	3.38	4.88
GPC (%)			10.40	10.99	11.07	10.48	10.83
GV			10.70	12.24	11.72	9.48	11.92
LWR			2.17	2.27	2.03	3.79	2.61
PC			light brown	red	light brown	light brown	light brown
subgroup			TEJ	TEJ	TEJ	TRJ	TRJ
SNP information							
AC	1	3,184,457	T	T	T	T	T
	1	3,329,456	A	A	A	A	A
	6	1,765,761	G	G	G	T	G
	6	20,854,368	A	A	A	G	G
	8	24,785,382	C	C	C	G	C
	12	17,068,981	G	G	G	G	G
GPC	11	11,666,233	A	A	A	A	G
	12	2,673,221	C	C	C	T	C
GT	2	20,220,266	T	T	T	A	T
	3	13,235,693	A	A	A	G	G
	3	16,312,643	C	C	C	C	C
	4	13,968,254	C	C	C	T	C
	4	24,196,718	A	G	G	G	G
	6	2,195,964	G	A	A	A	G
	6	6,687,883	T	C	C	T	T
	6	6,830,286	G	G	G	G	G
	6	7,024,152	T	T	T	C	C
	8	4,750,713	A	A	A	G	G
	8	20,763,355	G	G	G	G	G
GV	3	8,210,958	C	C	C	C	C
	3	16,733,441	G	G	G	T	T
	5	5,355,339	C	C	C	A	A
	6	12,504,994	C	T	T	C	C
	7	24,413,754	A	G	G	G	G
	12	21,773,741	T	G	T	T	G
LWR	3	5,210,399	A	A	A	A	A
	3	16,733,441	G	G	G	T	T
	3	16,749,510	C	C	C	C	C
	4	19,267,463	C	C	C	T	C
	4	24,917,693	T	T	T	T	T
	5	5,371,772	G	G	G	A	A
	5	28,480,050	G	G	G	G	G
	5	28,487,739	C	C	C	C	C
	7	217,125	T	T	T	T	T
	7	22,113,010	A	A	A	T	A
	7	25,214,651	C	C	C	T	C
PC	2	9,655,892	C	T	C	C	C
	4	20,157,178	C	C	C	C	C
	4	28,610,659	G	G	G	G	G
	7	6,068,017	C	C	C	C	C
	7	7,239,363	A	A	A	A	T
	7	10,922,526	C	C	C	C	C
	8	2,373,426	A	A	A	A	A

*GT, gelatinization temperature; AC, amylose content; GPC, grain protein content; PC, pericarp color; LWR, length/width ratio; and GV, grain volume*.

In this study, we performed GWAS to identify QTLs associated with GT, AC, GPC, PC, LWR, and PC, and many of them were overlapped with previously reported genes. The *qGT6-2* was only 60 kb from the *OsSSIIa* gene, and *qAC6-1* was located inside the *OsGBSSI* gene. *Rc* was also remapped in this study, associated with PC (*qPC7-1*). *GS3* (Fan et al., 2006) and *GW5* (Weng et al., 2008) were the well-studied genes regulating grain size, grain length, and grain width. These two genes were also detected related to LWR (i.e., *qLWR3-2* and *qLWR5-1*) and GV (i.e., *qGV3-2* and *qGV5-1*). Besides, *OsRPH1* was reported associated with plant height, grain length, width, and thickness in rice, which was covered by *qLWR5-3* in this study. In addition, the key missense SNPs were also identified in this study (Supplementary Table 5). Above all, we indicated the efficiency and accuracy of this study. Besides, only two QTLs were identified related to GPC. By comparing the GPC based on the SNP in the whole population, we found that both loci exhibited significant differences (*p* = 9.75E-05 for *qGPC11-1* and *p* = 0.0011 for *qGPC12-1*) for GPC (Supplementary Figures 1A,C). For S12_2673221, individuals who carried T exhibited higher GPC than those with T in both IND (*p* < 0.01) and TRJ (*p* < 0.001) subpopulations, which had similar trends in the whole population (Supplementary Figures 1C,D). While for *qGPC11-1*, the materials that carried G showed significantly higher GPC compared with those with A, which was opposite to the whole population. Thus, this site might be a unique QTL controlling GPC in the *Japonica* subspecies.

## Conclusion

This study has confirmed a wide variation of grain quality and appearance in the RDP1 accessions and identified a few germplasm lines superior to GT, AC, GPC, PC, LWR, and GV for rice grain quality and appearance improvement. A total of 19 and 24 loci associated with grain quality and appearance were identified, and nine of them were simultaneously identified by both the MLM and BLINK methods. Significantly, a candidate gene, *OsFbox394*, regulating the seed development was discovered in *qLWR7-2*, and *OsHyPRP45* was a candidate gene manipulating AC in rice grain. Further experiments are required to validate the function of these genes. Above all, this study provides basic information for further studies on genetic and molecular biology on grain quality and appearance in rice.

## Data Availability Statement

The original contributions generated for the study are included in the article/supplementary material, further inquiries can be directed to the corresponding author.

## Author Contributions

HZ and SL carried out the data analyses and wrote the manuscript. GZ, CZ, and ZP provided comments during the writing of the manuscript. ZW, JY, and YL revised the manuscript. All authors reviewed and approved the final manuscript.

## Conflict of Interest

The authors declare that the research was conducted in the absence of any commercial or financial relationships that could be construed as a potential conflict of interest.

## Publisher's Note

All claims expressed in this article are solely those of the authors and do not necessarily represent those of their affiliated organizations, or those of the publisher, the editors and the reviewers. Any product that may be evaluated in this article, or claim that may be made by its manufacturer, is not guaranteed or endorsed by the publisher.

## Supplementary Material

Supplementary Figure 1 Comparison of *qGPC11-1* in the whole population **(A)** and subpopulations **(B)**. Comparison of *qGPC12-1* in the whole population **(C)** and subpopulations **(D)**.

Supplementary Figure 2 Comparison of QTLs identified by MLM or BLINK carrying major and minor alleles of each SNP associated with the traits. **(A)** *qGT2-1*, **(B)** *qAC1-2*, **(C)** *qLWR3-1*, **(D)** *qLWR5-2*, and **(E)** *qLWR7-2*.

Supplementary Table 1 The sample list and structure information of O. sativa accessions.

Supplementary Table 2 Spearman's correlation among the traits.

Supplementary Table 3 LD blocks information for each QTL.

Supplementary Table 4 List of candidate genes located in the LD blocks.

Supplementary Table 5 Annotation of all missense SNPs located in candidate genes using SnpEff software.
