# Supplementary figures and images for: Genetic Diversity Relationship Between Grain Quality and Appearance in Rice
Source: Front Plant Sci. 2021 Aug 2;12:708996. doi: 10.3389/fpls.2021.708996 (PMC8365354; doi:10.3389/fpls.2021.708996)

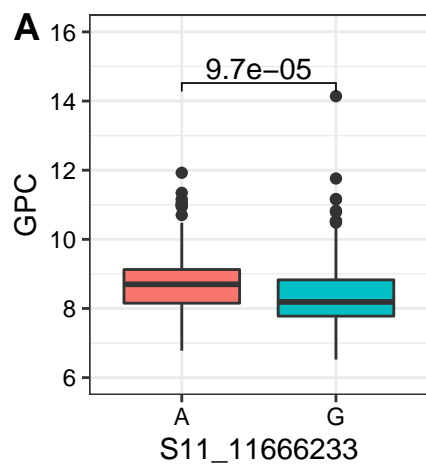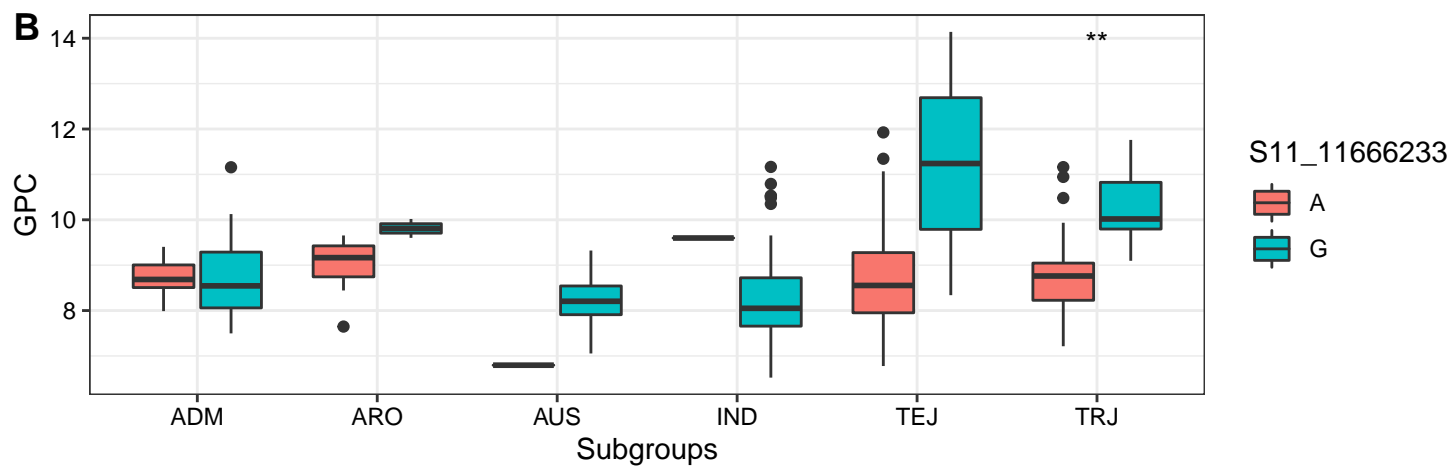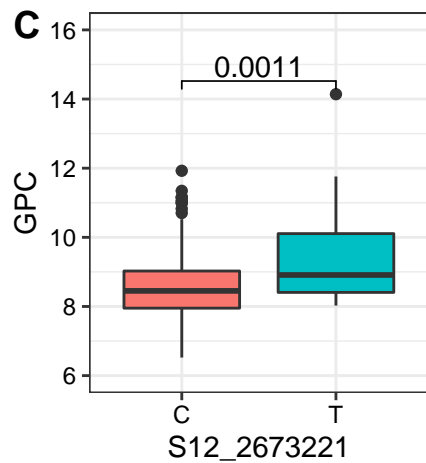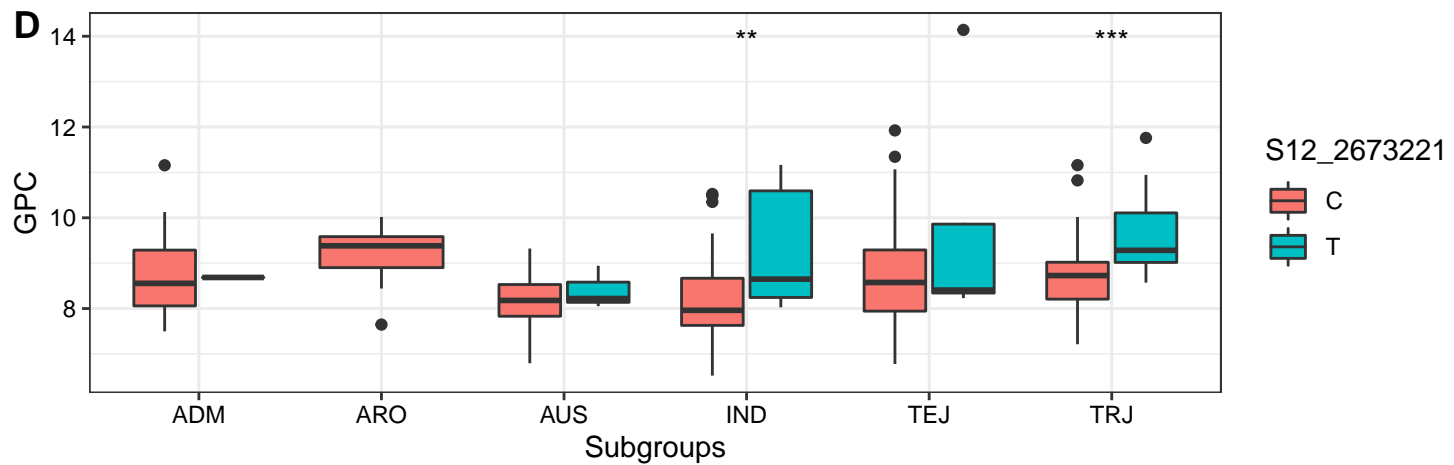

Supplement: Supplementary Figure 1 — Comparison of qGPC11-1 in the whole population (A) and subpopulations (B). Comparison of qGPC12-1 in the whole population (C) and subpopulations (D). [file Image_1.PDF]

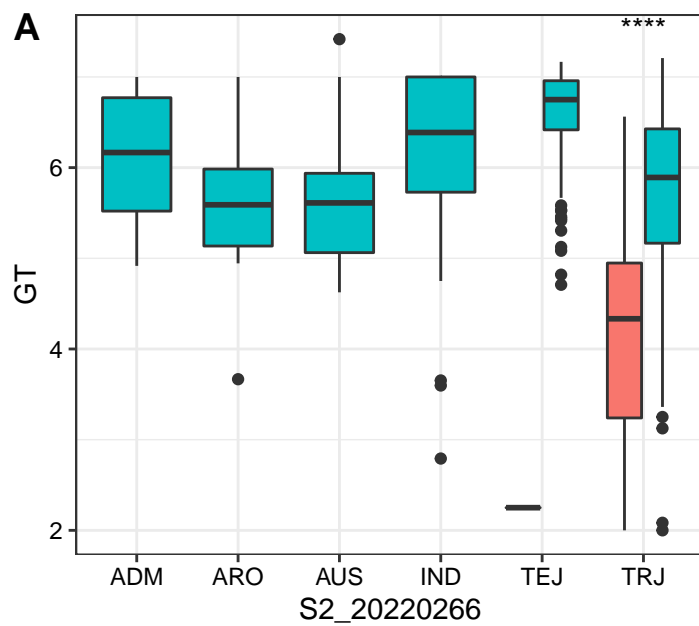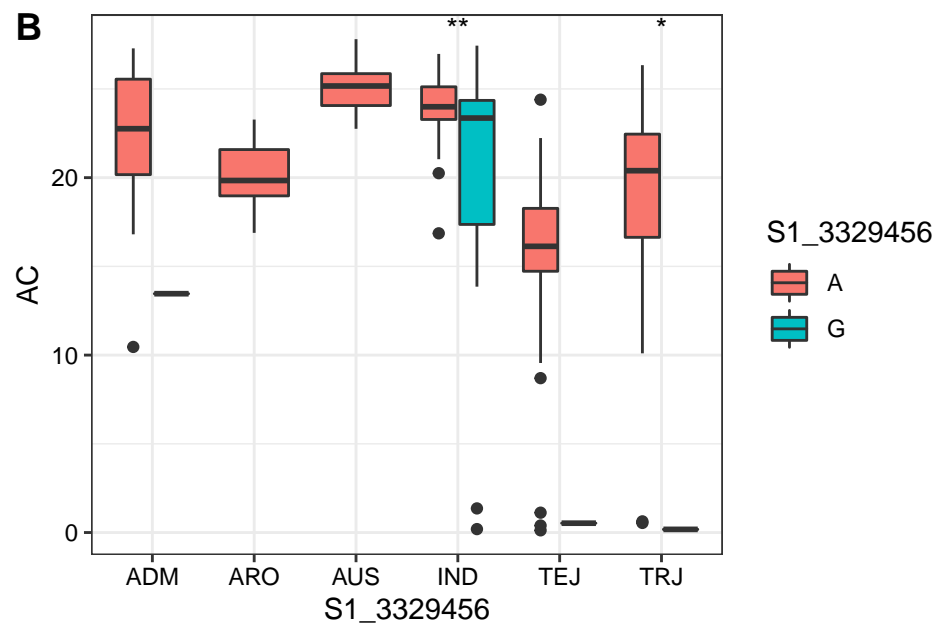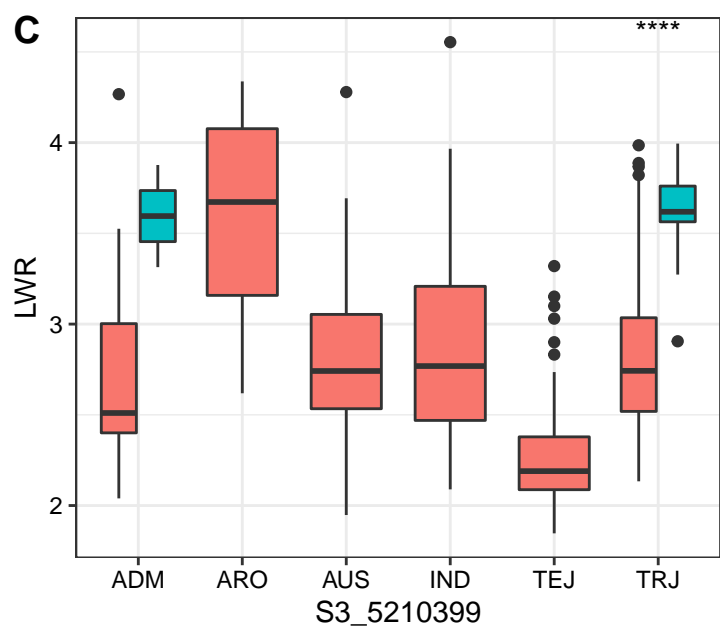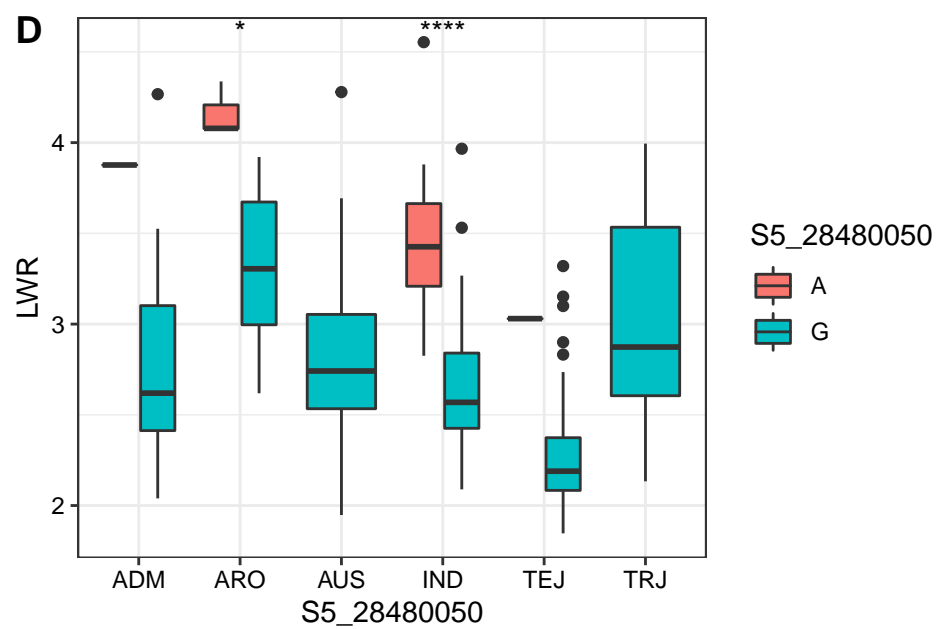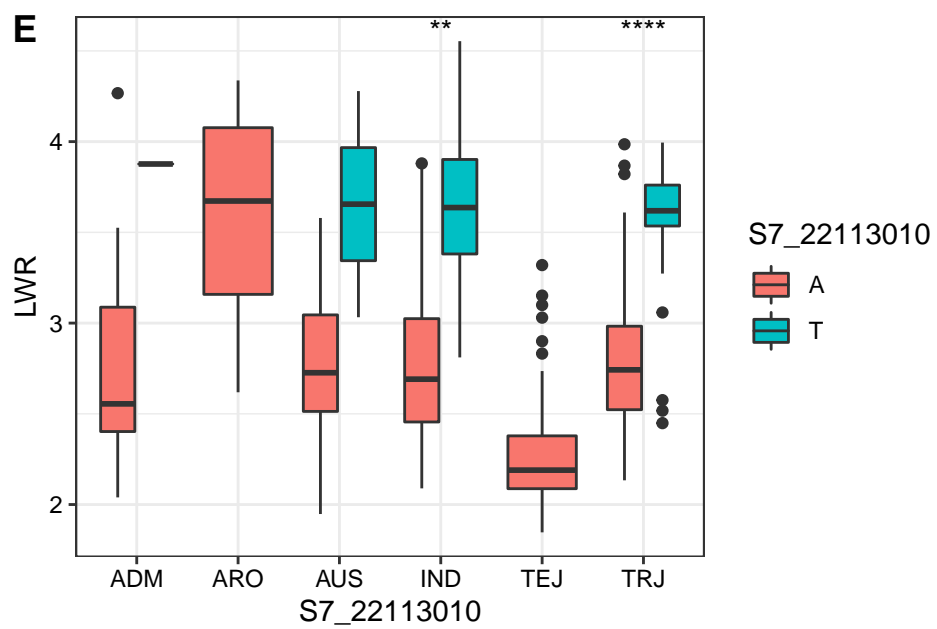

Supplement: Supplementary Figure 2 — Comparison of QTLs identified by MLM or BLINK carrying major and minor alleles of each SNP associated with the traits. (A) qGT2-1, (B) qAC1-2, (C) qLWR3-1, (D) qLWR5-2, and (E) qLWR7-2. [file Image_2.PDF]
